# Phytonutrient Profiles of Mistletoe and Their Values and Potential Applications in Ethnopharmacology and Nutraceuticals: A Review

**DOI:** 10.3390/molecules30224390

**Published:** 2025-11-13

**Authors:** Maeleletse G. Mopai, Semakaleng Mpai, Johannes Van Staden, Ashwell R. Ndhlala

**Affiliations:** 1 Department of Plant Production, Soil Sciences and Agricultural Engineering, University of Limpopo, Private Bag X1106, Sovenga 0727, South Africa; mopai.glas@gmail.com (M.G.M.); semakaleng.mpai@ul.ac.za (S.M.); 2 Research Centre for Plant Growth and Development, School of Life Sciences, University of KwaZulu-Natal, Pietermaritzburg, Private Bag X01, Scottsville 3209, South Africa

**Keywords:** bioactive compounds, ethnomedicinal, haustorium, hemiparasitic, *Loranthus*, phenolic composition, phytochemicals, *Viscum*

## Abstract

Mistletoe species grow hemiparasitically on a wide spectrum of trees occurring in the tropical, subtropical and temperate zones. Mistletoe has been associated with fertility, vitality and fatality in humans. In the present review, we examine articles published in English and/or with the option to translate them into English on the distribution mechanism, ecological impact and value. This study aimed to interrogate and outline the phytonutrient profiling, ethnomedicinal and ethnopharmacological applications, toxicology and quality control of mistletoes, particularly the Loranthaceae and Viscaceae families. There are at least 1400 species of mistletoe belonging to these two families across the world. Humans have used mistletoe for years, and it has a rich history, mainly in traditional medicine. Recent research interests accompanied by investigations on mistletoe have made a major contribution to our understanding of the survival behavior and mechanisms of the species. This has prompted researchers to intensively investigate and justify its potential and applications in traditional medicine systems to further incorporate it into conventional scientific medicine processes. This study thoroughly outlines the nutritional and phytochemical constituents of various mistletoe species, as well as the factors that play a role in the process. The sections in this study dedicated to elaborating on the nutritional and phytochemical composition of mistletoe show that it is a plant species rich in nutrients and bioactive compounds, especially compared to its host. Its survival mechanism makes it possible for this plant species to contain significant quantities of both nutrients (proximate components and mineral elements) and bioactive compounds (viscin, alkaloids, phenolics and polyphenolics, tannins, flavonoids, terpenoids, glycosides, and saponins). Overall, mistletoe species have potential as medicinal plants, offering a range of health benefits and the ability to treat ailments (both communicable and non-communicable diseases). However, there are insufficient investigations and records of mistletoe’s toxicity, safety and quality control. Therefore, it is important to further investigate the potential applications, mechanisms of action, and optimal uses of mistletoe preparations and/or extracts.

## 1. Introduction

Throughout history, humankind has relied on nature for its basic needs, such as the production of foodstuffs, shelter, clothing, means of transportation, fertilizers, flavors and fragrances, and, most importantly, medicines for the treatment of various ailments [1]. Plants have formed the basis of sophisticated traditional medicine systems that have been in existence for thousands of years and continue to provide humankind with new remedies [1]. Mistletoe species have played a significant role as a traditional herbal medicine. Mistletoe is a general term given to a group of aerial obligate parasitic angiosperms that are epiphytic to a wide range of host trees from several families and genera. Mistletoe comprises evergreen parasitic plant species that occur on the stems and/or branches/barks of woody plants, conifers and deciduous host trees [2]. They range from hemiparasites that photosynthesize but access nutrients and water from the host xylem to holoparasites that are not capable of photosynthesis and must therefore access photosynthetic products from the phloem of host trees [3]. They have evolved from root parasitic species, whereby, during evolution, they developed a special multi-functional organ known as an haustorium, which penetrates the shoot to connect to the vascular tissues of a host [4]. This parasitic-specific intrusive organ serves as an anchor and as a root for the absorption of water and minerals from its host [5,6,7,8]. For nutrient supply, this species strictly relies on the xylem sap of the host plant for the extraction of some pharmacologically significant metabolites, but, due to a lack of connection between the phloem tissues of the host and epiphyte, the flow of photo-assimilates is restricted from the epiphyte to the host. Haustorium development by mistletoes enables them to overcome direct resource competition from the soil with other more competitive plants, and, by becoming aerial, they minimize canopy shade [9].

Most mistletoe species are of a generalist nature, while some are specialist mistletoes. Polhill and Wiens [10] state that, in Africa, 70% of mistletoes are generalists, parasitizing a wide range of host families, 12% are specific to a few families but occasionally parasitize species of other families, and 18% are specific to host species from one genus. Mistletoe species are primarily represented by the most common, diverse and widespread families including Loranthaceae and Viscaceae, which are taxonomically related to each other, and share the order Santalales [11]. Loranthaceae comprises at least 75 genera with over 900 species, and about 7 genera and an estimated 500 species belong to Viscaceae [11]. Over the years, studies have focused on the common European mistletoe species *Viscum album*, which revealed much of what is known about this intriguing group of plants [12]. Only in recent years has research focused on other mistletoe species from various genera [13,14,15,16,17,18,19].

This study aimed to generate a comprehensive review of the phytonutrient profiling, ethnomedicinal values and applications, and toxicity and safety of some preparations and extracts of various mistletoe species that belong to the most common and diverse families, Loranthaceae and Viscaceae.

## 2. Methodology

Several electronic databases, including Google Scholar, ScienceDirect, PubMed, PubChem, Wiley Online, Scopus, Elsevier, and Springer Nature, were scrutinized. Articles, abstracts, and theses were also used as sources of information. The keywords included in the search were: “Loranthaceae”, “Viscaceae”, “systematics”, “Mistletoe ”, “seed dispersal”, “distribution”, “nutritional composition”, “bioactive compounds”, “host-parasite interactions”, “ecological impacts”, “physiological effects”, “ethnopharmacology”, “preparation”, “extract”, “Helix^®^”, “Iscador^®^”, “AbnobaViscum^®^”, “lectin”, “viscotoxin”, “cancer”, “oncology”, “clinical trial”, “therapy”, “safety”, “toxicology”, “pharmacology”, “commercialization”, “anthroposophic medicine”.

## 3. Distribution and Habitat of Mistletoe Families, Loranthaceae and Viscaceae

Mistletoe species are mainly distributed in major biomes of different climate types [20,21,22]. Mistletoes normally occur in tropical and subtropical regions of Africa, Asia, Australia and Madagascar as well as in temperate zones of Europe, southern Asia and Africa. They are rare and/or absent in parts of northern Africa and completely absent in extremely cold areas [4,23,24], including Russia, Canada and Alaska. Mistletoe distribution is greatly impacted by a wide spectrum of biotic and abiotic factors. Temperature is one of the most critical abiotic factors affecting the occurrence and/or life of mistletoes [25]. Summer and winter temperatures play a fundamental role in limiting the geographical distribution of mistletoe. For instance, the optimum temperatures conducive to mistletoe occurrence range between 15 and 20 °C [25]. Mistletoe has perennial leaves and photosynthetic stems, and it is very sensitive to low winter temperatures, which determines its cold tolerance, and thus its northernmost or uppermost distribution limits [26,27]. According to Varga et al. [28], the distribution limits of mistletoe (*V. album*) correlate with the monthly mean temperatures of the warmest and coldest months of the year. Furthermore, Varga et al. [28] stated that in the event of climate change, such as global warming, mistletoe is likely to benefit and expand its range. Iversen [26] also reported that mistletoes are limited in their geographic distribution by both low summer and low winter temperatures. Skre [29] found that the mean monthly temperatures of the coolest and the warmest months related closely to the heat sum that is needed as energy for the respiration and growth of evergreen plants (respiration equivalent; RE). For example, the temperature data for these 2 months outlines the limits of occurrence of *V. album* ssp. *album*. Besides climatic conditions, the global occurrence and distribution of mistletoe is governed by several factors such as preferred host tree availability, altitude and elevation, geographic location, vegetation type, bird dispersal, habitat disturbance and the presence of competing plants.

## 4. Ecological Impact and Value of Mistletoes

Mistletoes are known to cause significant damage to forests, orchards, plantations, and ornamentals worldwide. According to Sangüesa-Barreda et al. [23], mistletoes are considered one of the major increasing agents of global forest decline. Hawksworth [30] also noted that mistletoes could impair growth, lower host vigor, reduce wood quality and quantity, and predispose trees to be attacked by insects, disease, and fungi. In North America, dwarf mistletoes (*Arceuthobium*), which parasitize conifers, are responsible for most of the damage. Dwarf mistletoes (Viscaceae family) can affect their hosts in various ways, including reducing their vigor, growth rate, flowering and fruiting, and the quality and quantity of forest products produced. They can also predispose trees to attack or damage by insects and infection by fungi, which can result in the premature death of commercially valuable trees [31,32]. The semi-parasitic African mistletoe (*Loranthus micranthus* L.) also causes similar consequences for tree development, although it depends on water and nutrients from the host plant [33].

Mistletoe impacts lead to fatalities, promote the growth of epicormic shoots on host branches, resulting in numerous witches’-brooms (Hexenbesen), cause swelling and cankers on the trunk that may accumulate resin, and cause spike-tops. These factors, especially the massive witches’-brooms, increase the fire hazard by altering the quality, quantity, distribution, and flammability of fuel in the forest [34]. Estimates indicate that these species caused annual wood losses of about 500 million cubic feet in the United States (U.S.) over the previous decade [35] and 150 million cubic feet in British Columbia. In areas where adverse abiotic factors are concentrated, such as increased erosion, higher pollination levels, and frequent drought, hosts become more susceptible to mistletoe infestation [36]. Hardwood trees such as *Fagus sylvatica* L. are resistant to mistletoe infestation, although other species such as *Quercus* spp. and *Ulmus* spp. are rarely affected [37].

Despite their nature as parasites and having major economic impacts, mistletoes (Loranthaceae and Viscaceae) are increasingly recognized as keystone species that contribute disproportionately to ecosystem function and biodiversity. Mistletoe-host relationship often transcends parasitism, showing facultative mutualism whereby the host provides structural support for mistletoe while mistletoes reciprocate by nutrient enrichment of the soil through rapid litter decomposition [38,39]. Mistletoes are regarded as ecological engineers due to their ability to modify their physical environment through soil nutrient cycling, hydrology, and microclimatic conditions (atmospheric carbon dioxide (CO_2_) [40]. Additionally, their dense growth structures create significant habitats for birds and arthropod communities [41,42,43]. Despite representing a minor canopy constituent with regard to biomass, at the ecosystem scale, mistletoe enhances the ecosystem/biodiversity through principal mechanisms (1) augmented floral resource provision (nectar, pollen) and the provision of nutrient-rich foliage that support and maintain diverse faunal assemblages, (2) creation of microhabitats through their dense growth morphology and induced host canopy architectural alteration, and (3) subsidization of detrital food webs through enriched litterfall quality and quantity that accelerates nutrient cycling [44,45,46].

As semi-succulent shrubs and relatively to their hosts, mistletoes are rich in tissue nitrogen, phosphorus (P), and potassium (K) concentrations [36,45]; therefore, they serve as critical forage sources for herbivores, from browsing mammals to arthropods [47]. Canopies infected by mistletoe maintain lower temperatures and high humidity rates, creating a microclimate that provides thermal refugia for fauna during heat stress, especially due to accelerating climate change effects [48]. The nutrient-enriched litterfall generated by mistletoe stimulates and promotes facilitative interactions across ecosystems. For example, a study by Watson and Herring [48] showed avian richness declined by 36% due to the removal of mistletoes from the Eucalypt woodlands. Subsequently, during drought conditions, ground-foraging insectivores were significantly negatively affected [48]. In contrast, woodlands infected by mistletoes exhibited greater resilience by supporting two-fold the proportion of woodland-dependent species post-drought conditions compared to woodlands that are mistletoe-free [49]. The overall effects of mistletoe, ranging from trophic support to biogeochemical cycling, underscore mistletoe’s capacity and ability to regulate, modify and amplify ecosystem processes across spatial scales. The ecological significance of mistletoe was placed beyond its parasitic nature [50,51].

## 5. Nutritional Composition of Mistletoe Species

Generally, medicinal plants are rich in nutrients and substances that have the potential to be used for therapeutics or to synthesize useful drugs [52]. The nutritional constituents of mistletoes are mainly linked to their interactions with their ecosystem. Mistletoe species are autotrophs that entirely or partially rely on the host for water and nutrients [53]. Their nutritional levels are greatly influenced by the physicochemical status of the host because of their parasitic habit [3] as well as other factors, including species, host tree species, environmental conditions, plant age, plant part, and seasonal variation. The plants’ nutritional benefits are due to their chemical composition [54]. Previous investigations have reported mineral element composition of different mistletoe species (Table 1). Calcium (Ca), potassium (K), iron (Fe), magnesium (Mg), manganese (Mn), sodium (Na), zinc (Zn), phosphorus (P), and copper (Cu) amongst other elements [nitrogen (N), sulfur (S), selenium (Se), iodine (I), molybdenum (Mo), and boron (B)], were the major mineral elements reported in different investigations on different mistletoe species belonging to the families Loranthaceae and Viscaceae (Table 1). There is scanty literature on proximate component analysis, such as moisture content, ash, crude fat, total carbohydrate, protein, crude fiber, crude protein, nitrogen-free extract, dry matter, and organic matter of mistletoe, particularly in the Loranthaceae and Viscaceae families. However, there has been a recent interest in investigations aimed at screening and/or profiling the proximate components of various Loranthaceae and Viscaceae species (Table 2).

Parasitism of plants by other plants provides an exceptional opportunity for investigating correlative nutritional relationships. Host plants play a significant role in the quality and quantity of elemental constituents in mistletoe species. The difference in elemental composition as well as natural constituents of mistletoe growing on different host plants is probably due to the nature of the host and interaction with these hosts [18]. Theories have proposed that the higher transpiration rate of mistletoe than its host serves as a nutrient-gathering role since mistletoe has evolved to have a lower energetic investment in haustoria than would be the case for a free-standing plant that requires structural and conducting roots [66]. Mistletoe’s nutrient acquisition mechanisms enable it to accumulate greater foliar mineral concentrations relative to their hosts [67]. Thus, mistletoes contain greater foliar mineral concentrations than their hosts [67] as well as some natural components. This is not the case in all elements and some organic components. However, little is known about whether hemiparasitic mistletoes actively access nutrients from the host phloem [68]. Because N is highly phloem-mobile while Ca is a large molecule, and is phloem-immobile, an N:Ca ratio > 1 implies an active uptake from the host phloem and may suggest that water and nutrient acquisition are not tightly coupled [53,69,70,71].

The nature of the host plants confers differences in the elemental and proximate constituents of mistletoes due to interaction with their host plants. Several studies have reported the presence of various proximate components in various mistletoe species (Table 2). According to Nwoke et al. [18], *T. bangwensis* leaves exhibited lower concentrations of moisture content and total ash. In contrast, carbohydrates and proteins were present in appreciable amounts. Kim et al. [62] on the other hand reported that the leaves of a *V. album* var. *coloratum* were found to contain relatively high moisture, ash, fiber and carbohydrate contents, and low crude protein and fat content. Some of the findings in the literature [17,61,64,65] agree with a study conducted by Mnisi et al. [63] who found that carbohydrate was the predominant proximate component in both leaves and twigs of *V. verrucosum* followed by crude protein, crude fiber, crude ash and crude fat. The lower moisture content present in plant materials implies the potential long shelf-life characteristic since water content is an indicator of water activity and can be used as a component to assess the stability and susceptibility of the plant material to microbial contamination [72,73]. Furthermore, low total ash content indicates possible low total mineral elements content. High ash content indicates the presence of some mineral elements [74]. The presence of carbohydrates and proteins may be due to conglomerates of bioactive sugars, glycoproteins or proteins that contribute to the plant’s medicinal potency against specific diseases or stresses [75]. Mistletoe extracts with high fiber and low fat content are nutritionally beneficial and reduce the risks of cancer and heart diseases as they play a crucial role in lowering the levels of serum cholesterol [76]. Although there are several factors involved in the presence of proximate components of mistletoe species, there is a discrepancy in several studies profiling and quantifying the proximate components.

**Figure 1:**
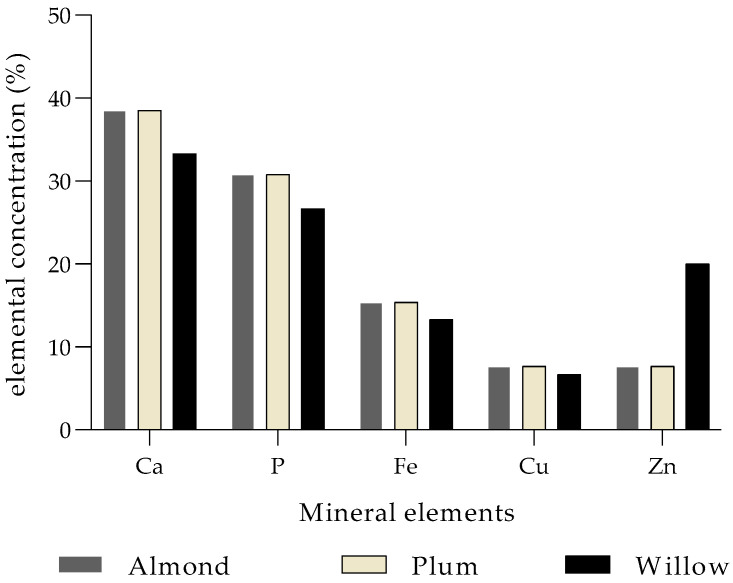
The effect of host tree on mineral element accumulation (%) in *V. album* [71].

Furthermore, mistletoes do not share the nutrients with their hosts to avoid mineral deficiency and cope with excess and imbalances [3]. A possible reason why many mobile nutrients are found in higher concentrations in mistletoes than in their host plants. Türe et al. [58] reported that the nutrient concentrations of N, P, K, Na, Zn, S and Cu; except Mo was higher in mistletoe (*V. album*) than its host, and Ca, Mg, Mn, Fe and B, lower in mistletoe than four different deciduous host trees, *Populus alba*, *Crataegus monogyna*, *Salix alba* and *Robinia pseudoacacia* (Table 3). In host trees, concentrations of Ca, Na, Zn, and Mo in *P. alba*; P, K, Mg, Mn, Cu, S, and B in *S. alba*; N in *R. pseudoacacia*; and Fe in *C. monogyna* are the highest compared to the concentrations recorded in mistletoes [58]. Umucalilar et al. [71] reported the presence of Ca, P, Fe, Cu and Zn in *V. album* at different vegetation stages, harvested from three different host species, inclusive of almond (*Amygdalus communis* L.), plum (*Prunus domestica* L.) and willow (*S. alba*) (Figure 1). The concentrations of both Ca and P were excessively higher in *V. album* from all three host species as compared to Fe, Cu and Zn [71]. The *V. album* harvested from almond (38.5%) and plum (38.5%) was predominated by Ca, and there were no significant differences. Cu and Zn were the lowest mineral elements present in *V. album* harvested from almond (7.7%) and plum (7.7%). However, there was an increased Zn percentage exhibited by the *V. album* from willow (20%). Samples of *V. album* harvested from willow plants exhibited the lowest percentage of Ca (33.3), P (26.7), Fe (13.3) and Cu (6.7) compared to *V. album* harvested from almond and plum (Figure 1). Elements such as Ca and B were higher in the host trees than in mistletoe, simply because of their limited mobility or immobile characteristics [77]. Lo Gullo et al. [66] also described the increase in mineral content in mistletoe compared to the host. Furthermore, Lo Gullo et al. [66], assumed that the lack of or limited photosynthetic activity in mistletoe leads to the accumulation of nutritional elements, mainly K and Ca. Several studies support these findings [77,78,79].

## 6. Phytochemical Composition of Mistletoe Species

Secondary metabolites, commonly referred to as phytochemicals, are non-nutrient natural plant bioactive compounds that possess the potential to mitigate disease due to their antioxidant activity [80]. Secondary plant metabolites are necessary for the survival of plants and help to establish interactions between plants and the environment [81]. Phytochemicals constitute an important part of the plant’s defense mechanism against pathogenic attacks and environmental stresses, in addition to providing a valuable range of natural products. Although natural products, particularly secondary metabolites, serve as the foundation for medications, their presence in plant biochemistry is frequently challenging to rationalize [1]. Mistletoe’s bioactive constituents include gastric-irritating alkaloids, cardiac toxins (viscotoxins, phoratoxins), lectins, saponins, sterols, terpenes, glycosides, lycopene, isoflavones, β-carotene [82], tannins, flavonoids, and phenolic compounds [83], yet the contents of these metabolites are unstable. Several studies have reported phytochemical constituents present in different mistletoe species from both the Loranthaceae and Viscaceae families (Table 4). Table 4 shows phytochemical screening of different mistletoe species reported in different studies [14,15,17,18,54,55,84,85,86,87,88]. However, alkaloids, anthraquinones, flavonoids, glycosides, saponins, tannins, steroids and terpenes appear to be the commonly studied and reported phytochemicals [14,15,17,54,55]. The most intriguing aspect is that some of these compounds are derived from the host tree rather than mistletoe. According to Martin Cordero et al. [83], some alkaloids can only be found in mistletoe growing on plants that synthesize these chemicals. Factors such as the region/location effect, host tree and seasonal variation have an impact on the chemical composition of mistletoe [89].

**Figure 2:**
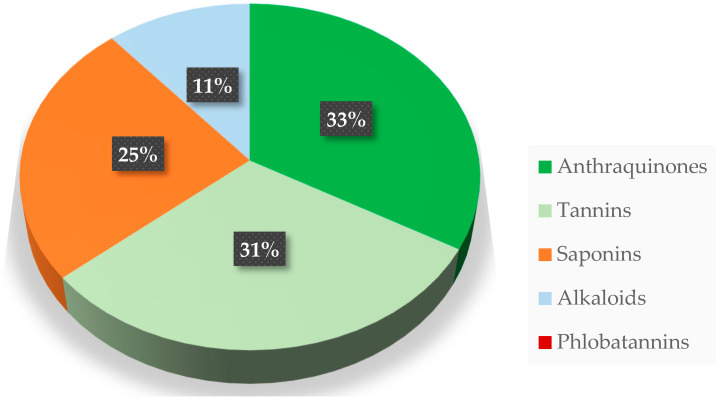
Phytochemical constituents’ concentrations (%) of the leaf extracts of African mistletoe (*T. dodoneifolius* (DC) Danser) [84].

Figure 2 illustrates the overall average phytochemical concentration (%) of African mistletoe’s (*T. dodoneifolius* (DC) Danser) leaf extracts harvested from 14 different host trees. Notably, there was a significant difference in terms of the presence of the phytochemical constituents of African mistletoe’s (*T. dodoneifolius*) leaf extracts tested [85]. Overall, the leaf extracts of *T. dodoneifolius* from 14 different host trees were predominated by anthraquinones (33%) and tannins (31%). Saponins (25%), followed by alkaloids (11%) were the least present phytochemical constituents of the *T. dodoneifolius* leaf extracts. In contrast, phlobatannins were absent (0%) in all the leaf extracts of *T. dodoneifolius* harvested from 14 distinct host trees (Figure 2). Due to its bioactive compounds and its beneficial effects on the human body, mistletoe has become more and more studied, developing various pharmaceutical formulations (Iscador, Isorel, Iscucin, Lektinol, Eurixor, Helixor, Abnoba-Viscum and recombinant mistletoe lectin (ML-1)) [24]. Several chemical and pharmacological studies have identified various types of compounds in mistletoe, such as lectins, viscotoxins, lignans, amines, flavonoids, and polysaccharides [90,91]. *S. atropurpurea* included 16 bioactive materials comprising six (6) fatty acid compounds, two (2) xanthines, two (2) flavonols, a glycoside, monoterpene glycosides, one (1) lignan glycoside, and four (4) flavones [92].

**Table 4:** Phytochemical screening of different mistletoe species belonging to Loranthaceae and Viscaceae families.

	Mistletoe	Phytochemicals Detected	References
Family Name	Scientific Name		
Loranthaceae	*Tapinanthus dodoneifolius* (DC.) Danser	Anthraquinones, a rare presence of alkaloids, saponins, and tannins	[85]
Loranthaceae	*Loranthus micranthus* L.	Alkaloids, saponins, tannins, flavonoids, glycosides and a few steroids	[89]
		Alkaloids, saponins, tannins, flavonoids, terpenoids, glycosides, reducing sugars	[86]
Loranthaceae	*Phragmanthera incana* (Schumach.) Balle	Anthraquinones, alkaloids, saponins, tannins, cardenolides	[54]
Loranthaceae	*Tapinanthus bangwensis* (Engl. & K.Krause) Danser	Flavonoids, saponins, tannins, and cardiac glycosides	[14]
		Steroidal glycoside, flavonoids, phenols, saponins, tannins	[18]
Loranthaceae	*Phragmanthera capitata* (Spreng.) Balle	Anthraquinones, alkaloids, phenolic acids, saponins, tannins, flavonoids, cyanogenic glycosides	[15]
Loranthaceae	*Loranthus micranthus* Hook.f.	Alkaloids, saponins, tannins, flavonoids, phenolic acids, steroids	[17]
Loranthaceae	*Tapinanthus preussii* (Engl.) Tiegh.	Anthraquinones, antioxidants, alkaloids, saponins, tannins, flavonoids, phenolic acids, steroids, cardiac glycosides, cyanogenic glycosides, carotenoids, phlobatannins	[55]
Loranthaceae	*Scurrula atropurpurea* (Blume) Danser	Polyphenols, tannins, flavonoids, monoterpenoids and sesquiterpenoids, steroids, triterpenoids and quinones	[84]
Viscaceae	*Viscum album* L.	Alkaloids, flavonoids, viscotoxins, lectins, phenolic acids, terpenoids, sterols, phenylpropanoids	[87]
		Terpenoids, fatty acids and vitamin E	[88]

Polyphenols are a group of bioactive compounds that possess significant functions in plants. There are approximately 8000 plant-derived polyphenols, and they are responsible for various metabolic processes. They have higher antioxidant power owing to their chemical structural characteristics [93]. The main polyphenols identified in mistletoes are from the phenolic acids class, both hydroxybenzoic acid (gallic acid, protocatechuic acid) and hydroxycinnamic acid (caffeic, ferulic acid, synaptic acid), and flavonoids including flavanone (naringenin, eriodictiol), flavone (apigenin), and flavonol (3-O-Methyl quercetin, myricetin, and kaempferol). Phenolic acids are important compounds not only for ensuring the construction of lignin, but also for the regulation of plant growth and disease resistance stimulation. For instance, hydroxy-cinnamic acids are associated with the role of plant growth regulators and proteins in the development of certain diseases. Flavonoids are a class of polyphenol compounds, secondary metabolites of vegetal origin, with antioxidant properties, which are found in the free state or seen as esters or glycosides in mistletoe [93]. Several studies have identified and reported polyphenols present in various mistletoe species (Table 5). Their presence and/or availability have been shown to be mainly influenced by the host tree and climatic conditions. However, the mechanisms remain controversial, and at present, most of the research focus is on either limited numbers of metabolic compounds or merely one of these two influencing factors mentioned above.

**Figure 3:**
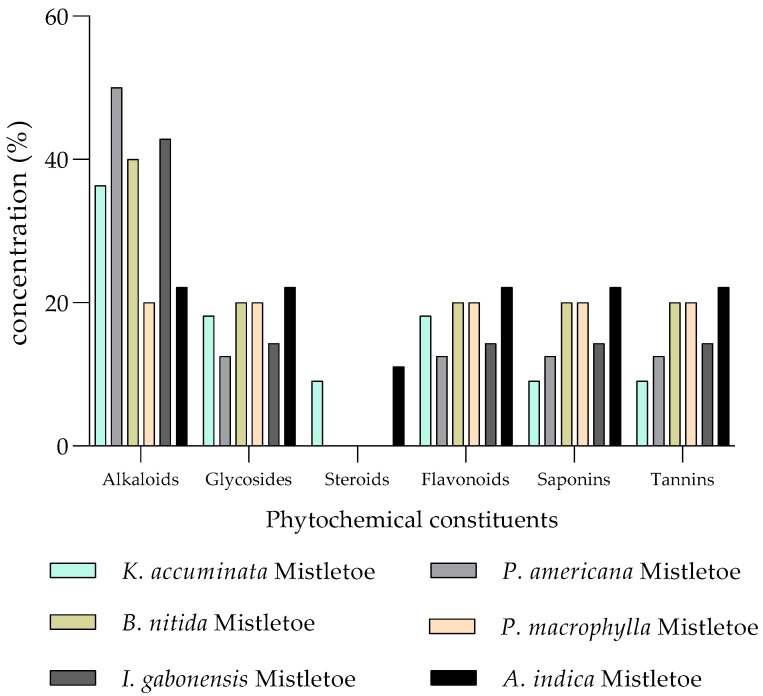
Screening and quantification (%) of phytochemical constituents of *L. micranthus* extracts harvested from six (6) different host trees [86].

Investigations have shown that the host tree species, its physiological state of development and harvesting period/season influence the phytochemical composition and antioxidant activities of mistletoes [86,98,99]. The preliminary results of a study conducted by Ukwueze et al. [86], June 2001 (winter season), showed the presence of bioactive compounds including tannins, alkaloids, saponins, flavonoids, glycosides and little or absence of steroids in all mistletoe (*L. micranthus*) extracts harvested from six different host tree sources, namely *Irvingia gabonensis*, *Pentaclethra macrophylla*, *Kola accuminata*, *Baphia nitida*, *Persea americana* and *Azadirachta indica* (Figure 3). The chemical constituents showed variation in terms of degree of content and not in terms of the kind of chemical compounds present in the extracts of mistletoes harvested from different host trees. For example, alkaloids were more predominant in mistletoe extracts of *L. micranthus* from *I. gabonensis*, *P. americana* and *B. nitida* than in the other three host tree species examined. Furthermore, glycosides and flavonoids were more predominant in mistletoe extracts harvested from *A. indica* than in the rest of the sources. Saponins were abundant in *A. indica* compared to other plant sources [86] (Figure 3).

## 7. Mechanistic Basis of Host Tree Influence on Mistletoe Biochemical Composition

The significant impact of the tree species hosting mistletoe on its chemical makeup, which includes its essential nutritional components like minerals, carbohydrates, and amino acids, along with unique phytochemicals, is foundational to mistletoe’s biology and poses a significant challenge for establishing uniform standards. This variation related to the host can be understood through a tiered series of linked processes, starting with how resources are gathered and continuing to how metabolism is controlled. The most straightforward mechanism involves mistletoe relying on the xylem sap of its host for all water and nutrient solutes. Each type of sap has a unique composition depending on the tree species, which leaves a fundamental chemical mark on the mistletoe parasite. The tree it attaches to directly influences the basic nutritional characteristics of mistletoe. This entails specific levels of both macro and micronutrients (for example, nitrogen, potassium, calcium, magnesium, zinc, copper) and the types of organic carbon and nitrogen compounds (such as specific amino acids like arginine and certain sugars) found in the xylem. For instance, a mistletoe growing on a pine tree located in nutrient-deficient soil will inherently have a different elemental and nutritional composition compared to one growing on an apple tree in nutrient-rich soil.

In addition to basic nutrition, the xylem sap could also contain particular organic precursors or signaling molecules (like jasmonic acid) that the mistletoe can either use or react to, which in turn can affect the pathways involved in creating complex secondary metabolites. The mineral content taken from the host not only adds nutritional value but also plays a crucial role in managing phytochemical creation. Various important minerals serve as cofactors for significant enzymes involved in secondary metabolic processes. For instance, the success of producing certain phenolic compounds or alkaloids can significantly depend on the presence of metal ions like copper or iron. Hence, a host that delivers xylem sap rich in certain micronutrients can enhance the mistletoe’s synthesis of specific bioactive compounds associated with those nutrients, establishing a direct connection between the nutrition derived from the host and the production of phytochemicals. A more intricate and less substantiated theory suggests a deeper manipulation by the host. It is possible that small regulatory RNA molecules or even functional genes could be transferred from the host to the mistletoe at their attachment site. This gene transfer could directly modify the expression of the mistletoe’s genes that control nutrient uptake, primary metabolism, or secondary metabolite production, resulting in a chemical profile that combines traits from both the mistletoe and its host.

In summary, the influence of the host tree is a multifaceted process. It begins with shaping the elemental and nutritional components of mistletoe through its xylem sap. This initial input lays the groundwork for developing a specific phytochemical profile by supplying the required substrates, cofactors, and potential signaling molecules that direct the mistletoe’s metabolic functions. Understanding these mechanisms illustrates why the choice of host tree is not merely a botanical aspect but a crucial factor determining the chemical and medicinal effectiveness of mistletoe extracts.

## 8. Ethnomedicinal Values and Application of Mistletoe

Plants have played a crucial role in preserving human health and well-being for millennia. In recent years, there has been renewed interest in natural medicines that are obtained from plant parts or plant extracts [100]. Medicinal plants are used globally as a valuable source for new drug formulations. A significant portion of the global population continues to depend on medicinal plants and other conventional remedies for their day-to-day healthcare needs [1]. Communities using traditional methods may not fully comprehend the scientific justifications for relying on indigenous treatments, but they are aware of the therapeutic benefits of certain plant extract doses [1]. There is a general belief amongst consumers globally that herbal drugs are always safe because they are “natural”. However, scientific evidence suggests otherwise. The mere fact that a product is “natural” may not signify that the product is safe [101]. Although limited evidence suggests that adverse effects associated with the use of herbal drugs are less likely to occur than with conventional drugs, they do usually occur mildly and only affect a small number of people. Recent evidence suggests that some of the herbs considered safe over the last many decades have proven to be associated with health hazards [101]. Historically, natural products or plants represent a valuable and rich source of biologically active compounds with recognized potential in drug discovery and development [102] to create safe, effective, and cost-efficient medications [103]. The therapeutic potential of plants is primarily attributed to their phytochemical components, which accumulate in various plant tissues such as roots, rhizomes, stems, leaves, flowers, or bark [56], which have shown several biological and pharmacological activities. Research on mistletoe in Africa has primarily focused on crop protection, with little attention given to its potential applications in ethnopharmacology [104]. There has been a growing interest in employing medicinal plants, including mistletoe, in developing countries due to the reported safety and lack of adverse effects associated with herbal medicines compared to synthetic drugs [14]. The use of natural health products derived from plants is increasing worldwide. It is known that approximately 80% of the population in developing countries relies on traditional medicine, which mainly consists of herbal prescriptions [105].

Mistletoes, traditionally valued medicinal plants, were described as “an all-purpose herb” due to its rich traditional uses [106] and has been widely used in ethnomedicinal fraternities for various purposes such as antihypertensive, anticancer, antispasmodic, antidiabetic, epilepsy, asthma, headaches, high blood pressure, menopausal symptoms, infertility, dermatitis, arthritis, and rheumatism [107]. However, these claims and/or investigations are rarely supported and validated by clinical studies. In vitro and in vivo studies confirm a broad spectrum of the therapeutic action of mistletoe species. There is an obvious discrepancy between the popularity of mistletoe extracts and their classification as a non-conventional treatment modality with unproven efficacy in oncology. The commercial preparations suffer from several major drawbacks: lack of precise declarations for the molecular characteristics and the concentrations of diverse extract constituents; the composition of extracts can even be influenced by the different methods of preparation, the time of harvest, and the type of host tree; lack of experimentally substantiated instructions for the dose of supposedly effective substance(s) and the schedule of applications to clinically trigger an indisputably documented antitumoral activity; lack of thorough clinical studies according to the generally accepted criteria as the measure for responsible recommendations [108].

Table 6 summarizes the pharmacological activities of different mistletoe species from the Loranthaceae and Viscaceae families. The ethnomedicinal value of these plants (mistletoe) is related to their phytochemical constituents. These bioactive compounds are commonly associated with pharmacologically fundamental activities. Whilst the mechanism of action of some of these bioactive compounds remains vague, there is, however, consensus that their wide pharmacological applications are due to their structural diversity. Mistletoe (*Viscum album*) preparations contain active components like mistletoe lectins (ML) and viscotoxins, and are reported to show antitumoral properties by causing cell cycle delay or arrest and induction of apoptosis [109,110], affecting tumor angiogenesis [111,112] and exerting immune-potentiating activities that may enhance the host defense system against tumors [113,114,115,116]. Molecular compounds of mistletoe are reported to show in vitro inhibitory potential on P-glycoprotein (P-gp), also known as multidrug resistance protein 1 (MDR1) [117]. Bioactive compounds, such as saponins, flavonoids, tannins, and cardiac glycosides extracted from mistletoe species have been shown to exhibit medicinal and physiological activities [118]. Flavonoids exhibit antibacterial, anti-inflammatory, antiallergic, antimutagenic, antiviral, antineoplastic, antithrombotic, and vasodilatory activities [119]. Flavonoids and phenolic acids are natural antioxidants involved in the biological activity of the plant and used in the prevention of diseases such as cancer, caused by oxidative stress induced by free radicals in the body. Tannins reported in a study by Omoruyi and Onyeneke [14] have a wide variety of applications, ranging from antiviral, antibacterial, antiparasitic, and inhibition of HIV replication in infected H9 lymphocytes with little toxicity, like epigallitannins. Cancer therapy with *V. album* extracts has been performed in Europe for over six decades on thousands of patients. Plant extracts are mainly used as injections. Currently, several mistletoe preparations (e.g., Helixor and Iscador) are used in many countries to treat different types of cancer [120]. Ohashi et al. [88] reported that the alkynic fatty acid octadeca-8, 10, 12-triynoic acid found as chemical constituents of *Scurrula atropurpurea* (Loranthaceae), a parasitic plant of the tea plant *Thea sinensis* (Theaceae), exhibited a potent inhibitory effect on cancer cell invasion in vitro.

## 9. Ethnopharmacological Correlations: Bridging Traditional Use and Scientific Validation

The customary applications of different mistletoe species, especially in European and African traditional medicine, are now being validated by contemporary pharmacological research to discover the active compounds and how they work. This scientific confirmation enhances the justification for additional research and possible advancement of therapies based on mistletoe. Table 7 outlines the main connectedness between the traditional applications and scientific validation of mistletoe extracts.

The common traditional use of *Viscum album* (European mistletoe) in cancer care is justified by the strong cell-killing and immune system-regulating effects of its main components, mistletoe lectins (MLs) and viscotoxins. ML-I has been shown to trigger cell death in several cancer cell lines and to activate immune cells, including natural killer (NK) cells, macrophages, and T-lymphocytes [124]. Viscotoxins demonstrate a direct ability to destroy the membranes of tumor cells [125]. These mechanisms offer a scientific foundation for using mistletoe extracts as additional treatment in cancer care. The conventional use of plants such as *L. micranthus* (African mistletoe) for treating high blood pressure is backed by proof of its effects on the cardiovascular system. Flavonoids and various phenolic compounds extracted from these species have demonstrated vasorelaxant properties on isolated aortic rings, as well as inhibitory activity against angiotensin-converting enzyme (ACE) in laboratory settings [126]. These activities are linked to a reduction in blood pressure in animal studies. The application of mistletoe for inflammatory ailments such as arthritis is supported by the anti-inflammatory properties of its flavonoid and phenolic acid constituents. Research indicates that extracts from mistletoe can block important pathways that cause inflammation, which includes the creation of cytokines such as TNF-α and IL-6, as well as enzymes like cyclooxygenase-2 (COX-2) [127]. Historical accounts of using mistletoe for diabetes are associated with its possible blood sugar-lowering effects. Extracts from *V. album* have been demonstrated to block carbohydrate-digesting enzymes such as α-amylase and α-glucosidase in laboratory studies, and they enhance glucose tolerance in animal models. These effects are believed to result from the presence of polysaccharides and flavonoids (Table 7) [128].

**Table 7:** Ethnopharmacological correlations of various mistletoe species.

Mistletoe Species	Traditional Use/Ailment	Putative Active Compound(s)	Reported Biological Activity (In Vitro/In Vivo)	References
*Loranthus micranthus*, *Viscum* *album*	Hypertension	Flavonoids (e.g., quercetin) and phenolic Acids	Vasorelaxant effects, ACE-inhibitory activity, antioxidant activity	[125,126]
*Viscum album*	Cancer/Tumors	Mistletoe lectins (MLs) and viscotoxins	Induction of apoptosis in cancer cells, immunomodulation (e.g., increased NK cell activity, cytokine release), direct cytotoxic/cytolytic effects	[124]
*Viscum album*, *Phragmanthera* spp.	Inflammation, arthritis	Flavonoids and phenolic acids	Inhibition of pro-inflammatory cytokines (TNF-α, IL-6), COX-2 enzyme inhibition, reduction in oxidative stress	[127]
*Viscum album*	Diabetes	Polysaccharides and flavonoids	Alpha-glucosidase and alpha-amylase inhibitory activity, improved glucose tolerance	[128]

## 10. Toxicology and Safety of Mistletoe Preparations: A Critical Analysis of Evidence and Gaps

According to Briskin [129], there has been an unwavering increase in the use and application of products derived from medicinal plants and scientific studies. Medicinal plants are envisioned to generate potent bioactive compounds crucial for enhancing human health and well-being. Although remarkable success has been achieved in the detection, treatment and management of diseases using conventional medicine, many patients still resort to orthodox medicine since many of them are believed/reputed to offer a complete cure with less serious side effects [129]. Synthetic drugs usually consist of a single chemical, while medicinal plants can contain complex mixtures of 400 or more chemicals. It is comparatively easy to figure out the activity and side effects of a single chemical, but there is no way scientists can map all the complex interactions and synergies that might be taking place between the various chemicals found in a plant, or crude plant extract containing these chemicals which is used traditionally [101]. However, most of these plants have been ingested indiscriminately without considering their toxicological adversities and safety. The advancement of technology has enabled scientists to detect minute amounts of carcinogenic and toxic chemicals in herbs and recognize or evaluate potentially hazardous effects of some of the herbs that have been used in traditional medicine for centuries [101]. The growing use of medicinal plants like mistletoe is driven by the perception of being “natural” and safer than synthetic drugs. However, the complex phytochemical nature of plant extracts makes a comprehensive toxicological profile challenging to establish. While mistletoe preparations have a long history of use, a critical analysis reveals significant gaps in our understanding of their safety, particularly regarding long-term toxicity, dose-dependent effects, and the robustness of clinical trial data. Subsequently, there are insufficient preclinical, in vitro and/or in vivo studies on the application, toxicity and safety of mistletoe preparations and/or extracts.

Existing preclinical and clinical studies primarily report that mistletoe extracts, such as Iscador^®^ and Helixor^®^, are well-tolerated in the short term [130,131,132]. The most commonly documented adverse effects are mild and localized, including reactions at the injection site, subfebrile temperatures, and transient eosinophilia [133,134]. Studies by Huber et al. [135] and Bergmann et al. [136] confirm that these effects are dose-dependent, linked to the key active compound, mistletoe lectin (ML). According to Huber et al. [132,133], repetitive and continuous subcutaneous injections of ML containing mistletoe products are known to cause blood eosinophilia, and allergic reactions are known as possible side effects (Table 8). The long-standing human use, spanning over 80 years, provides a degree of clinical reassurance against acute, severe toxicity [129]. Although there seems to be short-term safety, the existing toxicological information is inadequate for a conclusive assessment of safety. The main shortcoming is the absence of thorough, long-term toxicology research. The majority of early research studies concentrated on immediate harmful effects. Important questions that still need answers are: “What are the overall effects of long-term administration beneath the skin on organ systems, particularly the liver and kidneys? Are there potential risks related to the immune system from prolonged changes to its activity, especially in individuals who do not have cancer or those with weakened immune systems?“ Moreover, the harmful effects of mistletoe compounds and the likelihood of interactions between herbs and drugs have not been thoroughly studied. This is a significant lapse, considering that cancer patients who use mistletoe as an additional treatment are probably also taking other medications, which could lead to interactions that might change the effectiveness or harmfulness of either treatment. While over 40 clinical trials have been conducted, their findings on efficacy remain “controversial” [130] largely due to methodological weaknesses that also impede a clear safety analysis. Many trials are limited by small sample sizes, short duration periods, and a lack of rigorous adverse events monitoring protocols focused on subjective, mild symptoms. A critical gap is the absence of large-scale, prospective, long-term cohort studies specifically designed to detect rare but serious adverse events. Table 8 provides the heterogeneity of preparations, dosages, and standardization methods [108]. While useful, it highlights another issue and/or existing challenge. This variability makes it nearly impossible to perform meaningful cross-study comparisons or meta-analyses to establish a reliable dose-toxicity relationship. For instance, the safety profile of a recombinant ML preparation [136] may not be directly comparable to that of a whole fermented extract such as Iscador^®^.

Current literature indicates that mistletoe extracts possess a favorable short-term safety profile within a limited dosage range. Nevertheless, this should not be interpreted as a lack of danger. The notable research deficiencies identified are the absence of long-term toxicological data, insufficient exploration of herb-drug interactions, and the methodological constraints of current clinical trials, which underscores an urgent necessity for a new wave of focused study. Future research should emphasize meticulously conducted toxic kinetic studies and comprehensive, large-scale clinical trials that systematically assess safety as a key outcome. Therefore, the promise of mistletoe therapy can only be balanced with a complete and scientifically valid comprehensiveness of its risks through such robust investigations.

## 11. Commercial Status of Mistletoe Preparations and Regulatory Environment

The development of mistletoe research to produce marketable products is mainly appearing in Europe, where these solutions are effectively incorporated into cancer treatment, especially in anthroposophic medicine. Nonetheless, the process of commercialization encounters obstacles, such as challenges in the widespread cultivation for decorative uses and issues related to quality assurance due to contamination in herbal products. Mistletoe is partially commercialized; however, prominent products such as Iscador^®^ (Weleda AG, Switzerland), Helixor^®^ (Helixor Heilmittel GmbH, Germany), and AbnobaViscum^®^ (Abnoba GmbH, Germany) are produced in accordance with rigorous quality control standards and are officially acknowledged as pharmaceuticals with designated regulatory classifications [137].

Within the European Union (EU), mistletoe products are generally classified as “anthroposophic medicinal products” in accordance with Directive 2001/83/EC [138]. This status recognizes their historical traditional usage. For certain purposes, like enhancing the quality of life and lessening symptoms in cancer patients, some products have received approval due to clinical evidence [139]. The production method is consistent, and every batch undergoes strict testing for biological effects (such as lectin levels) and safety [138]. Anthroposophic preparations play a significant role in mistletoe therapy, surpassing mere branding to reflect a unique philosophical approach and production method. These preparations are developed with a focus on the host tree’s specific traits, affecting their medicinal value. They undergo a fermentation process that modifies their toxicity and boosts immune effects. Unlike standard pharmaceuticals, these products are standardized based on the manufacturing process. They also have a long history of clinical use in integrative medicine.

In the United States (US), the regulatory environment is significantly different. Mistletoe extracts do not have approval from the Food and Drug Administration (FDA) to be used as prescription medications for cancer therapy. They are, however, accessible as dietary supplements according to the Dietary Supplement Health and Education Act (DSHEA) of 1994. This classification indicates that they cannot be legally sold with statements suggesting they can diagnose, treat, cure, or prevent any illness. As a result, their application in cancer treatment is regarded as “off-label” and is typically not included in coverage by most insurance policies. Clinical research in the US is currently active, with a focus on producing strong data needed for possible FDA approval as an additional treatment option [139].

There are factors associated with commercialization and applications of mistletoe preparations, but not limited to, global markets and challenges. The worldwide market for mistletoe products is specialized but steady, influenced by demand from Central Europe [124]. Significant obstacles to broader commercialization include regulatory challenges, the complexity of standardization and scientific dispute. The biological activity varies greatly depending on the host tree, the time of harvest, and the method of extraction, presenting a significant challenge for standardizing production between batches. Obtaining authorization as a prescription medication in new markets such as the US necessitates extensive, randomized controlled trials that are both expensive and time-intensive [140]. The continuing discussion about clinical effectiveness, as mentioned earlier, still acts as an obstacle to the broad approval by the established oncology community [141].

## 12. Conclusions and Recommendations

In the current study, a few of the many mistletoe species belonging to the families Loranthaceae and Viscaceae have been intensively explored and reported on. Their distribution and occurrence, nutritional and phytochemical composition as well as ethnomedicinal potential and applications have been recognized. The general link between parasitic infection and increased tree mortality rates is well established and the modifications of host processes following mistletoe infection are increasingly well understood. Most studies on the phytonutrient profiling of mistletoe are comparative investigations focused on the effects of mistletoe-host interaction and seasonal variations. Additionally, many in vitro studies are aimed at assessing the effects of solvent types and extraction techniques on the phytonutrient profiling of mistletoe. These gaps need to be filled through robust research.

It is evident that mistletoe species play a vital role in maintaining and improving human health, due to their nutritional and phytochemical constituents. The quantities of these nutritional and phytochemical constituents are highly influenced by both biotic and abiotic factors, with the host tree playing a major role. The impact of the host tree starts at the most basic level by determining the diet of the mistletoe through the makeup of the xylem sap. This directly influences its nutritional worth. Following this, the distinctive blend of nutrients, along with possible signaling molecules, generates a particular biochemical setting that influences the production and storage of its specialized phytochemicals. Grasping these processes is crucial for the growth and standardization of mistletoe extracts, as selecting the appropriate host tree significantly influences the chemical properties and healing capabilities of the resulting product. Mistletoe species, both from the Loranthaceae and Viscaceae families, have been shown to exhibit significant concentrations of a wide range of bioactive compounds with strong antioxidant activity that can be employed in ethnomedicine for the treatment of a variety of communicable and non-communicable ailments. Mistletoe extracts have shown that they can be used for treating various ailments; however, only a few of these scientific findings are supported and validated by clinical studies. Therefore, there is a need to continue to investigate, through robust clinical studies, the potential applications and uses of mistletoe extracts to approve and validate the non-clinical claims. Moreover, intensive research is imperative and needed to fully comprehend the effects (short- and long-term) and optimal uses of mistletoe extracts. Ensuring the efficacy and safe use through quality control and standardization of mistletoe extracts and/or products is equally crucial. There is scant literature covering the direct effects of biotic factors such as climatic conditions and habitat structure and heterogeneity, and abiotic factors including host-health and vigor, and water and nutrient availability on the nutritional and phenolic compositions of mistletoe species. Therefore, the climate-mistletoe, climate-host and mistletoe-host health interactive effects on biochemical compounds (nutrients and phytochemicals) need to be investigated and documented.

## Figures and Tables

**Table 1:** Mineral elements present in different mistletoe species belonging to Viscaceae and Loranthaceae families.

Mistletoe Species		Elements	References
Family	Scientific Name		
Loranthaceae	*Tapinanthus preussii* (Engl.) Tiegh.	Ca, Fe, K, Na, Mg, Zn, PO_4_^3−^	[55]
Loranthaceae	*Tupeia antarctica* (G.Forst.) Cham. & Schltdl.	Ca, Mg, K, Na, P, N	[56]
Viscaceae	*Viscum album* L.	Ca, Mg, K, P	[57]
Viscaceae	*Viscum album* L. subsp. *album*	Ca, Mg, Fe, Mn, N, P, K, Na, S, Cu, Zn, B	[58]
		Ca, N, P, K, Na, S, Mg, Fe, Cu, Zn, Mn, Mo, B	[59]
Loranthaceae	*Ileostylus micranthus* (Hook.f.) Tiegh.	Ca, Mg, K, Na, P, N	[60]
Loranthaceae	*Loranthus micranthus* Hook.f.	Ca, Mg, P, Zn	[17]
Loranthaceae	*Tapinanthus bangwensis* (Engl. & K.Krause) Danser	Ca, Mg, Mn, P, Zn, K, I, Fe, Na	[18]
Loranthaceae	*Phragmanthera incana* (Schumach.) Balle	Ca, Mg, K, Na	[54]
Viscaceae	*Viscum album* subsp. *austriacum* (Wiesb.) Vollm.	Ca, Mg, P, K, Mn, Cu, Zn, Fe, Se, S, Cl	[16]
Viscaceae	*Viscum articulatum* Burm. f.	Ca, Mg, Mn, Cu, K, P, Fe, Zn, N	[13]

**Table 2:** Components present in various mistletoe species.

Family Name	Botanical Name	Nutritional Constituents and Est. Value (g/100 g Dry Weight)	References
Loranthaceae	*Loranthus micranthus* Hook.f.	Moisture (18), crude fiber (10.5), crude protein (11.2), nitrogen-free extract (69.5), fat (3.8) and ash content (4.5)	[17]
Loranthaceae	*Tapinanthus bangwen-* *sis* (Engl. & K. Krause) Danser	Crude fat (4.2), protein (12.5), total carbohydrates (68.4), crude fiber (9.8), moisture content (7.5) and ash (5.1)	[18]
		Moisture (8.2), ash (4.8), crude protein (14.1), ether extract (3.5), crude fiber (11.2) and nitrogen-free extract (NFE) (66.4)	[61]
Loranthaceae	*Tapinanthus preussii* (Engl.) Tiegh.	Moisture (7.8), ether extract (fat) (4.5), ash (5.5), protein (13.8), crude fiber (10.8) and carbohydrates (65.4)	[52]
Viscaceae	*Viscum album* var. *coloratum*	Carbohydrates (58.4), crude protein (16.8), crude fiber (14.2), crude ash (5.8) and crude fat (4.8)	[62]
Viscaceae	*Ziscum verrucosum* (Harv.)	Dry matter 927.3, organic matter 821.6, crude protein (123.4), neutral detergent fiber, acid detergent fiber and acid detergent lignin	[63]
Loranthaceae	*Phragmanthera incana* (Schumach.) Balle	Fat (5.1), moisture (6.9), crude fiber (12.4), crude protein (15.3), ash (6.2) and carbohydrates (61.0)	[64]
Viscaceae	*Viscum album* L.	Crude protein (12.33), crude oil (5.49), crude ash (7.39), neutral detergent fiber (31.37), and acid detergent fiber (25.73)	[65]

**Table 3:** Mineral element screening and level of abundance in mistletoe species (*V. album*) harvested from four different host trees [58].

Elements	Host Tree Species			
	*P. alba*	*S. alba*	*R. pseudoacacia*	*C. monogyna*
N	+↓	+↓	++↑	+↓
Ca	++↑	+↓	+↓	+↓
P	+↓	++↑	+↓	+↓
K	+↓	++↑	+↓	+↓
Na	++↑	+↓	+↓	+↓
Mg	+↓	++↑	+↓	+↓
Mn	+↓	++↑	+↓	+↓
Fe	+↓	+↓	+↓	++↑
Zn	++↑	+↓	+↓	+↓
Cu	+↓	++↑	+↓	+↓
S	+↓	++↑	+↓	+↓
Mo	++↑		+↓	+↓
B	+↓	++↑	+↓	+↓

++↑ indicates the abundant presence of nutrients higher in the host tree than in mistletoe; +↓ indicates the presence of lower nutrients in the host tree than in the mistletoe.

**Table 5:** Polyphenols detected in different mistletoe species.

Family Name	Scientific Name	Phenolic Acids	Flavonoids	References
Viscaceae	*Viscum album* L. ssp *album*	Gallic acid (12.5 mg/g), caffeic acid (3.2 mg/g), ferulic acid (1.1 mg/g), p-Coumaric acid (0.8 mg/g), salicylic acid, gentisic acid, grotocatechuic acid, p-hydrobenzoic, rosmarinic acid, and sinapic acid (other acids detected in trace amounts)	3-O-Methyl quercetin (8.7 mg/g), apigenin (2.1 mg/g), and naringenin (0.5 mg/g)	[94]
Viscaceae	*Viscum album* L. ssp *abietis*	p-Coumaric acid (9.8 mg/g), ferulic acid (4.5 mg/g), caffeic acid (2.1 mg/g), salicylic acid, protocatechuic acid, 4-hydrobenzoic acid, vanilic acid, and sinapic acid (other acids detected in trace amounts)	Rhamnetin (5.5 mg/g), 3-O-Methyl quercetin (3.2 mg/g), naringenin (1.1 mg/g), rhamnazin, and apigenin (other acids detected in trace amounts)	[95]
Viscaceae	*Viscum album* L. ssp *austriacum*	Sinapic acid (15.2 mg/g), ferulic acid (6.7 mg/g), caffeic acid (3.4 mg/g), protocatechuic acid, salicylic acid, 4-hydroxybenzoic acid, vanilic acid, and p-Coumaric acid (other acids detected in trace amounts)	Quercetin (10.1 mg/g), myricetin (9.5 mg/g), kaempferol (4.3 mg/g), naringenin, eriodictyol, sakuranetin, isorhamnetin, rhamnazin, and rhamnetin (other acids detected in trace amounts)	[96]
Viscaceae	*Viscum album* L. ssp *coloratum*	Not detected (ND)	Eriodictyol (0.8 mg/g)	[97]

**Table 6:** Ethnomedicinal values/importance of various mistletoe species.

	Mistletoe Species	Medicinal Values and Application	References
Family Name	Scientific Name		
Loranthaceae	*Loranthus bengwensis* L.	To treat *Diabetes mellitus*	[98]
Loranthaceae	*Tapinanthus dodoneifolius* (DC.) Danser	Antimicrobial activities against certain multiple drug-resistant bacteria and fungal isolates	[81]
Viscaceae	*Viscum album* L. var *album*	Diabetes, high blood pressure and insomnia	[54]
Viscaceae	*Viscum album* L.	To treat skin diseases and prostate cancer, anticancer, antihypertensive activity and antidiabetic properties	[120]
Loranthaceae	*Tapinanthus bangwensis* (Engl. & K.Krause) Danser	Antihypertensive and antidiabetic agents	[121]
		Hypotensive, hypoglycemic and hypolipidemic effect, which lowers blood pressure, blood glucose and lipid profile	[14]
Viscaceae	*Viscum articulatum* Burm. f.	Paste and decoction are given to cure cuts, wounds, bone fractures, ulcers and blood diseases, epilepsy and sprains	[122]
Loranthaceae	*Loranthus ferrugineus* Roxb. ex. Jack	Hypertension and gastrointestinal complaint management	[123]
Viscaceae	*Viscum album* L. var. *coloratum* Ohwi	Material for anticancer functional foods for treatment of tumorigenic cells	[109]

**Table 8:** Applications of different dosages of different mistletoe preparations in clinical trials.

Preparation	Extract	Host Tree	Application	Dosage (mg Extract or ng ML)	Standardization
Phytotherapeutic preparations					
Eurixor^®^	Aqueous (herb)	Poplar	Subcutaneous, intracutaneous, intravenous	1 mg or 70 ng/ampule (1 mL)	ML-I
Lektinol^®^	Aqueous (herb)	Poplar	Subcutaneous	0.02–0.07 mg or 15 ng/ampule n(0.5 mL)	ML-I
Anthroposophic preparations					
Helixor^®^	Aqueous (herb)	Apple, fir and pine tree	Subcutaneous	0.01–50 mg/amp. (1 mL), 100 mg (2 mL)	Process
Iscador^®^	Aqueous lacto-fermented (herb)	Elm and oak tree	Subcutaneous	0.0001–20 mg/ampule (1 mL)	Process
Isorel^®^	aqueous (planta tota)	M, P, A	Subcutaneous, intramuscular	1–60 mg	Process

Note: M, P, A stand for Maple, Pine, Apple, respectively.

## Data Availability

Not applicable.
